# Supplementary material for: Nanopore fingerprinting of supramolecular DNA nanostructures
Source: Biophys J. 2022 Aug 18;121(24):4882–91. doi: 10.1016/j.bpj.2022.08.020 (PMC9808562; doi:10.1016/j.bpj.2022.08.020)
Supplement: Document S2. Article plus supporting material [file mmc3.pdf]

Biophysical Journal
Article

Nanopore fingerprinting of supramolecular DNA nanostructures

Samuel Confederat,[1,2] Ilaria Sandei,[3] Gayathri Mohanan,[1,2] Christoph Wälti,[1,2,*] and Paolo Actis[1,2,*]
[1]School of Electronic and Electrical Engineering and Pollard Institute, University of Leeds, Leeds, United Kingdom; [2]Bragg Centre for Materials Research, Leeds, United Kingdom; and [3]School of Chemistry, University of Leeds, Leeds, United Kingdom

ABSTRACT DNA nanotechnology has paved the way for new generations of programmable nanomaterials. Utilizing the DNA origami technique, various DNA constructs can be designed, ranging from single tiles to the self-assembly of large-scale, complex, multi-tile arrays. This technique relies on the binding of hundreds of short DNA staple strands to a long single-stranded DNA scaffold that drives the folding of well-defined nanostructures. Such DNA nanostructures have enabled new applications in biosensing, drug delivery, and other multifunctional materials. In this study, we take advantage of the enhanced sensitivity of a solid-state nanopore that employs a poly-ethylene glycol enriched electrolyte to deliver real-time, non-destructive, and label-free fingerprinting of higher-order assemblies of DNA origami nanostructures with single-entity resolution. This approach enables the quantification of the assembly yields for complex DNA origami nanostructures using the nanostructure-induced equivalent charge surplus as a discriminant. We compare the assembly yield of four supramolecular DNA nanostructures obtained with the nanopore with agarose gel electrophoresis and atomic force microscopy imaging. We demonstrate that the nanopore system can provide analytical quantification of the complex supramolecular nanostructures within minutes, without any need for labeling and with single-molecule resolution. We envision that the nanopore detection platform can be applied to a range of nanomaterial designs and enable the analysis and manipulation of large DNA assemblies in real time.

SIGNIFICANCE Solid-state nanopores have enabled the single-molecule detection of a range of analytes but often lack the ability to provide quantitative information of heterogeneous samples. Here, we demonstrate a single-molecule and high-throughput approach for the analysis of higher-order DNA origami assemblies with a nanopore. The technique enables the characterization of DNA origami nanostructures at statistically relevant numbers in real time and at single-molecule resolution while being non-destructive and label-free and without the requirement of lengthy sample preparations or the use of expensive reagents. Furthermore, we quantify the assembly yield of DNA origami nanostructures based on their equivalent charge surplus computed from the ion current signals recorded.

INTRODUCTION

The use of DNA as a building block for the engineering of nanoscale materials is one of the corner stones of DNA nanotechnology (1). In particular, the invention of the DNA origami approach, which exploits Watson-Crick base pairing between a single-stranded DNA scaffold and multiple short staple strands to fold the scaffold into a specific predefined geometry (2), allowed the folding of nanostructures with large surface area while simultaneously enabling spatially controlled assemblies and site-specific chemical functionalization (3). These unique characteristics have enabled the assembly of nanostructures and patterns with controlled geometry and function, either by directly folding the scaffold (4,5) or via higher-order assembly of pre-formed DNA tiles (6–8), which found applications in biosensing (9–13), drug delivery (14,15), and as tools for studying biological processes (16,17), *inter alia*.

Fueled by these rapid developments in engineering and fabricating complex DNA nanostructures, there is an increasing demand for their characterization, including the assessment of assembly yields. Traditionally, the characterization and assessment of yield of DNA constructs has relied on methods such atomic force microscopy (AFM), which relies on multiple scans of the folded structures and counting molecules from each scan, and agarose gel electrophoresis, which relies on measuring the intensity of the separated bands associated with each DNA nanostructure (18). A

Submitted June 23, 2022, and accepted for publication August 16, 2022.
*Correspondence: c.walti@leeds.ac.uk or p.actis@leeds.ac.uk
Editor: Tamar Schlick.
https://doi.org/10.1016/j.bpj.2022.08.020
© 2022 Biophysical Society.
This is an open access article under the CC BY license (http://creativecommons.org/licenses/by/4.0/).

rapid, label-free, and single-molecule approach would complement these techniques and would provide a valuable addition to the existing tools.

In recent years, nanopores have been established as a powerful tool for the characterization of DNA nanostructures with single-molecule resolution. Nanopore sensing is based on the measurement of transient perturbations of the ion current through a nanopore caused by the translocation of an analyte (19,20). The characteristics of these perturbations, such as the amplitude and duration, provide information about the physico-chemical properties of the translocating molecule, including size, charge, and shape (21,22). Nanopore sensing has also been successfully applied for the detection of colloidal nanoparticles (23,24) and biological nanoparticles including virus particles (25), ribonucleic particles (12), protein aggregates (26), and DNA nanostructures (27–30). Our group has previously demonstrated the analysis of two-dimensional DNA origami (31) and single-molecule biosensing (13) using nanopores and has recently shown the marked enhancement of single-molecule detection within a crowded nanopore (32). Other groups used DNA-based nanoswitches for sensing applications using nanopores (33), and a similar approach has been used for miniaturized molecular data storage (34).

Here, we report the single-molecule detection of supramolecular DNA assemblies by solid-state nanopore analysis. We demonstrate that the perturbations in the ion current caused by the translocation of a DNA origami nanostructure can fingerprint different states of higher-order assemblies, ranging from an individual monomer building block to multimer assemblies. We quantify the assembly yields of a range of higher-order assemblies of DNA nanostructures with single-entity resolution and benchmark the nanopore analysis against agarose gel electrophoresis and AFM imaging.

MATERIALS AND METHODS

DNA nanostructures

The design of the four DNA nanostructures used here follows the design published by Tikhomirov et al. (6,35) and was carried out using the CaDNAno 2.3 software (36). All nanostructures were folded from the single-stranded DNA M13mp18 (7249 bp) following the standard procedure for DNA origami fabrication (4). For the higher-order assemblies, the individual tiles were first folded individually using three main kinds of staples: bridge staples, interior staples, which constitute the main body of the tile, and a specific set of edge staples, which allow for a specific interaction between the structures (the complete list of the staples is provided in the supporting material). While the same set of bridge staples and interior staples is shared by all DNA nanostructures, each one of them has a different set of edge staples (Fig. S15). The edge staples can be "giving" (featuring a two-nucleotide extension), "receiving" (with a two-nucleotide truncation), or inert (characterized by two hairpins, forming a loop conformation, and preventing the further higher-order assembly of DNA origami along that side). Each structure can have eight giving/receiving staples or five inert staples. For the assembly, we followed published protocols (6,35) where the edges of each tile can be labeled as north, south, east, and west. Each set of interactive edge staples is complementary to just one other specific set of edge staples (north giving edges are complementary to west receiving edges, west giving edges to south receiving edges, east giving edges to north receiving edges, and south giving edges to east receiving ones), allowing for a targeted assembly and avoiding spurious interactions. Furthermore, the addition of the negation strands, which are complementary to the edge staples, inactivate the excess edge staples so that the monomers can be used in higher-order assemblies (6,35). To fold the structures, the individual monomer structures having complementary edge sequences were mixed together in equal concentrations and volumes, in order to increase the yield of the assembly. Specifications on the sequences and the interactions between the pre-assembled monomers for all the higher-order structures used in this work are provided in the supporting material.

DNA nanostructure folding

The single-stranded M13mp18 DNA scaffold 7249 bp was purchased from New England Biolabs (NEB; Hitchin, UK) at an initial concentration of 250 μg/mL. The staple strands were purchased from Integrated DNA Technologies (IDT, London, UK) and resuspended in 1× TE buffer pH 8 (Sigma Aldrich, Gillingham, UK) to a final concentration of 100 μM. The negation strands were purchased from IDT and resuspended in 1× TE buffer (pH 8) to a final concentration of 200 μM. For the folding, the single-stranded DNA scaffold (final concentration of 10 nM) was mixed with the staple strands (final concentration of 75 nM) in 1× TE buffer (pH 8) with 12.5 mM of $Mg(Ac)_2$ in 80 μL total volume. To fold the individual tiles, the solution of scaffold and staples was heated to 90°C for 2 min and annealed using a temperature ramp from 90°C to 20°C at 6 s per 0.1°C in a Mastercycler Nexus PCR Thermal Cycler (Eppendorf, Hamburg, Germany). Following the annealing step, the negation strands were added to the solution to achieve a final concentration of 375 nM in 80 μL final total volume. Another temperature ramp was then applied, going from 50°C to 20°C at 2 s per 0.1°C. The folded nanostructures were purified using Sephacryl S400 (GE Healthcare, Chalfont Saint Giles, UK) size-exclusion columns in order to remove the excess staple strands, and the product was eluted in 1× TE (pH 8) with 12.5 mM $Mg(Ac)_2$. The concentration of the individual tiles was measured using a NanoDrop 2000c Spectrophotometer (Thermo Fisher Scientific Inchinnan, UK) to prepare solutions with equal concentration. To fold the higher-order assemblies, equal volumes of the required individual monomers were mixed in PCR tubes and annealed using a temperature ramp from 55°C to 45°C (at 2 min per 0.1°C) followed by a ramp from 45°C to 20°C (at 6 s per 0.1°C). The folded nanostructures were imaged by AFM to confirm the successful assembly.

AFM imaging

For AFM imaging, 10 μL of purified DNA sample diluted to a final concentration of 0.5 nM in 1× TE (pH 8) with 12.5 mM $Mg(Ac)_2$ was deposited on a freshly cleaved mica substrate (Agar Scientific, Stansted, UK) and incubated at room temperature for 15 min. An additional 150–180 μL of 1× TE (pH 8) with 12.5 mM $Mg(Ac)_2$ buffer was added to the sample to facilitate the imaging. The samples were imaged using a Dimension Fastscan Bio (Bruker, Coventry, UK) in tapping mode in liquid with Fastscan D Si_3N_4 cantilevers with a Si tip (Bruker). We used the following imaging parameters: scan rate = 2–8 Hz, 256 samples/line, amplitude setpoint = 150–300 mV, drive amplitude = 3000 mV, integral gain = 1, proportional gain = 5. The data were processed using Nanoscope analysis 1.9.

Agarose gel electrophoresis

The quality of the DNA origami nanostructures was further inspected with agarose gel electrophoresis (Fig. S2). For this, 50 mL 0.7% agarose gel was

prepared using 1× TAE buffer containing 12.5 mM $Mg(Ac)_2$. The DNA origami samples were prepared by mixing a 20 μL aliquot at a concentration of 10 ng/μL with 4 μL of 6× Tri Track Loading Dye (Thermo Fisher Scientific). M13mp18 scaffold was added as a reference. A GeneRuler 1 kb DNA double-stranded ladder (Thermo Fisher Scientific) was used as molecular marker and positive control (Fig. S2). The running buffer consisted of 1× TAE and 12.5 mM $Mg(Ac)_2$. The gel was run at a constant voltage of 70 V for 120 min. The gel was then stained for 30 min with Diamond Nucleic Acid Dye (Promega, Southampton, UK) diluted in 1× TAE buffer. For this, 5 uL 10,000× concentrated Diamond Nucleic Acid Dye was diluted in 1× TAE buffer, and the gel incubated at room temperature for 30 min on a rocking platform. The agarose gel imaging was carried out using the GeneSnap software. Quantification of the gel bands for each DNA origami sample was done using ImageJ.

Nanopore fabrication and characterization

The nanopores were fabricated starting from 1.2 × 0.9 mm quartz capillaries (QF120-90-10; World Precision Instruments, Hitchin, UK) with the SU-P2000 laser puller (Sutter Instrument, Novato, CA, USA), using a two-line program: (1) heat, 800; filament, 4; velocity, 30; delay, 145′; pull, 95 and (2) heat, 730; filament, 3; velocity, 40; delay, 135; pull, 150. The pulling parameters are instrument specific and lead to nanopipettes with a nanopore diameter of approximately 160 nm. The nanopipettes were characterized by measuring their resistance in 0.1 M KCl (~40 MΩ), and the nanopore dimensions were confirmed by scanning electron microscopy using a Nova NanoSEM at an accelerating voltage of 3–5 kV (Fig. S1).

Nanopore translocation measurements

The translocation experiments were carried out by filling the nanopipette with the translocation buffer (100 mM KCl, 0.01% Triton-X, 10 mM Tris, 1 mM EDTA [pH 8.0]) containing the DNA origami sample at a concentration of 500 pM. The nanopipette was then immersed in a 100 mM KCl bath with the addition of 50% (w/v) polyethylene glycol (PEG) 35 kDa (ultrapure grade, Sigma Aldrich). An Ag/AgCl wire (0.25 mm diameter, GoodFellow, Huntingdon, UK) was inserted in the nanopipette barrel and acted as the working electrode, while a second Ag/AgCl wire was immersed in the bath and acted as the reference electrode. The DNA origami nanostructures were driven from inside the nanopipette into the external bath by applying a negative potential to the working electrode placed inside the nanopipette with respect to the reference electrode in the bath. The ion current was recorded with a MultiClamp 700B patch-clamp amplifier (Molecular Devices, San Jose, CA, USA) in voltage-clamp mode. Data were acquired at a 100 kHz sampling rate with a 20 kHz low-pass filter using the pClamp10 software (Molecular Devices). The ion current traces were further analyzed with the MATLAB script Transanlyser, developed by Plesa et al. (37). The obtained translocation events were analyzed by applying a 7-sigma threshold level from the baseline, and only the events above the threshold were considered translocation events (Fig. S6). The obtained events were further analyzed and plotted using Origin 2019b.

RESULTS AND DISCUSSION

DNA nanostructures assembly

In this study, we demonstrate the discrimination of supramolecular DNA nanostructures with solid-state nanopores by exploiting the unique signature in the translocation signal resulting from the ion current perturbation when the nanostructures pass through the nanopore (Fig. 1).

We assembled four different DNA nanostructures, starting from a pre-assembled DNA origami (85 × 85 nm) used as a building block (referred to hereafter as monomer), followed by a two-monomer assembly (dimer), a three-monomer assembly in an L-shape (trimer), and lastly, a 2 × 2 array of monomers (referred to hereafter as 2 × 2) (Fig. 2 *A*). AFM measurements confirmed the correct folding of the DNA nanostructures (Fig. 2 *B*).

Nanopore analysis of DNA origami monomer

We chose a nanopore diameter of 160 nm to be large enough to accommodate the largest DNA nanostructure (2 × 2) while retaining a sufficient signal-to-noise ratio for the detection of the monomer. The detection of our smallest

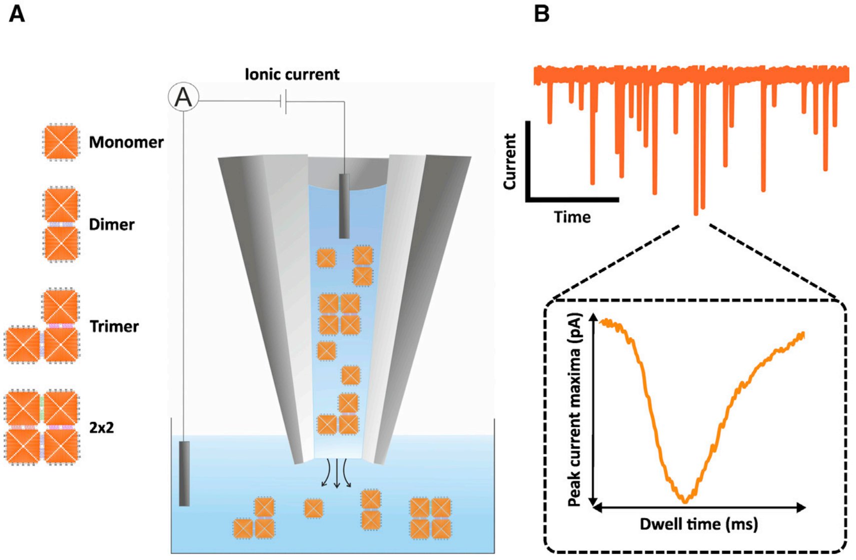

FIGURE 1 (*A*) Schematic representation of the nanopore setup. The DNA origami nanostructures are translocated from inside a nanopipette into the outer bath upon application of a negative potential bias while the ion current is measured. (*B*) Representative ion current trace showing translocation events. A representative event is shown in the inset with the translocation peak characteristics. Representative dwell time and peak current maxima values for different extracted translocation peaks are shown in Fig. S11. To see this figure in color, go online.

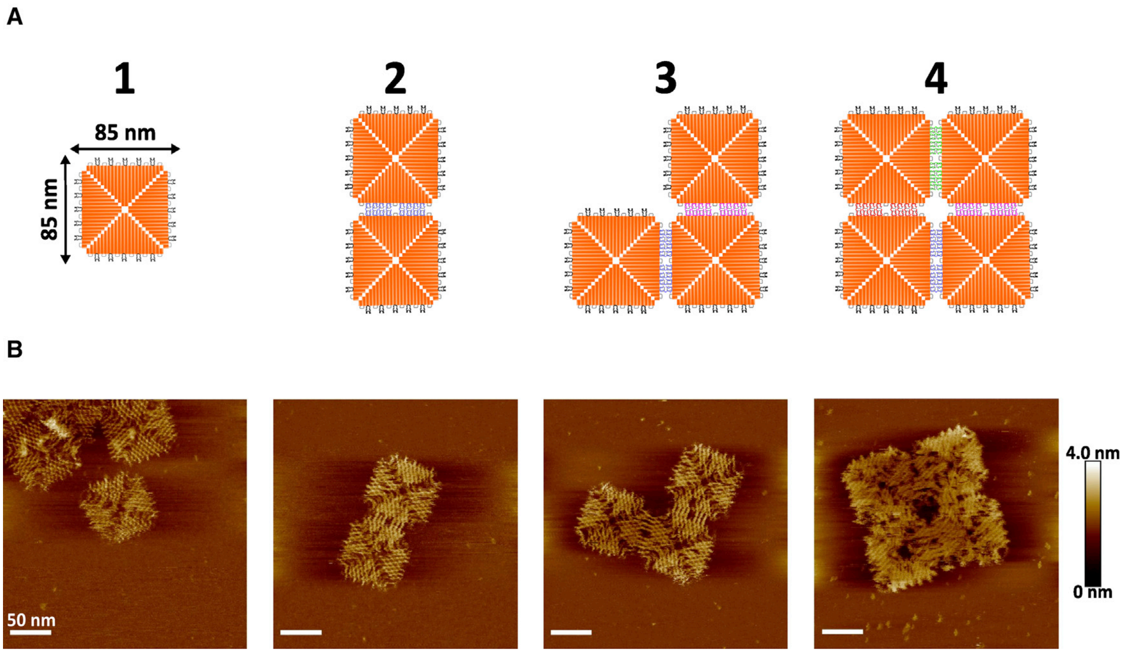

FIGURE 2 (*A*) Schematic representation of the DNA nanostructures, starting with a single monomer building block (*1*), dimer (*2*), trimer (*3*), and 2 × 2 (*4*). (*B*) AFM micrographs of the DNA nanostructure depicted in (*A*) (50 nm scale bar). To see this figure in color, go online.

(monomer) and largest (2 × 2) DNA nanostructures with a fixed pore size was facilitated by adapting the bath conditions where the nanopipette is immersed. We have previously demonstrated the marked enhancement in single-molecule-detection sensitivity when the commonly used macromolecular crowder PEG is added to the bath solution to a final concentration of 50% w/v (32,38). The presence of PEG resulted in translocation peaks that were well-resolved from the ion current baseline (Fig. 3 *A*).

In contrast, the translocation of monomer DNA nanostructures in the absence of PEG resulted in a low signal-noise-ratio, constraining the detection of the monomer nanostructures (Fig. S4). Furthermore, the presence of PEG allowed us to detect all DNA nanostructures with a high signal-noise-ratio and to record stable ion current traces with high capture rate (∼5 events/s at −300 mV) for several minutes without any evidence of nanopore clogging (Fig. S5). We first investigated the translocation of the monomers (Fig. 3 *B*) from inside the nanopipette into the outside bath under a constant voltage bias of −300 mV (Fig. 3 *A*). The translocation of the monomer DNA nanostructures resulted in conductive (current-enhancing) translocation signal peaks, where each peak is associated with the passage of one molecule. No peaks are detected under the same conditions if no analyte was added to the nanopipette (Fig. S7 *A* and *B*). The translocation events of the monomer sample can be characterized using the peak current maxima (maximum amplitude of the peak from the baseline) and dwell time (duration time of an event). The results ($N = 2307$ peaks) are displayed in the density scatter plot of Fig. 3 *C*, which shows that the translocation peaks fall within a well-defined area. Density scatter plots are commonly used in nanopore experiments to display the characteristic signature of the analyte (39). The histograms showing the peak current maxima and the dwell time distributions, respectively, are also shown in the figure. Both distributions have been fitted with a Gaussian distribution function yielding an average peak amplitude of 123 ± 27 pA and an average dwell time of 2.1 ± 0.6 ms. Upon increasing the applied voltage, we observed an increase in the peak current maxima of the conductive peaks, as well as a decrease of the dwell time (Fig. S8 *A*–*D*), suggesting that the DNA nanostructures are electrophoretically driven through the pore. This observation was also confirmed by translocation controls where no potential was applied (Fig. S7 *D*) or the applied bias was reversed (Fig. S7 *C*). Furthermore, we also probed the effect of DNA nanostructure concentrations and the effect of applied voltage on the translocation event frequency. With an increasing concentration of DNA nanostructures, more molecules are expected in the capture region of the nanopore, which results in a larger number of translocation events. We confirmed this observation by running translocation measurements at different concentrations (50–500 pM), as shown in Fig. S8 *F*. Moreover, increasing the applied voltage induces a larger capture region, leading to a greater number of translocation events (Fig. S8 *E*). These observations are consistent with established physical models describing the translocations of nucleic acids through solid-state nanopores (40–42).

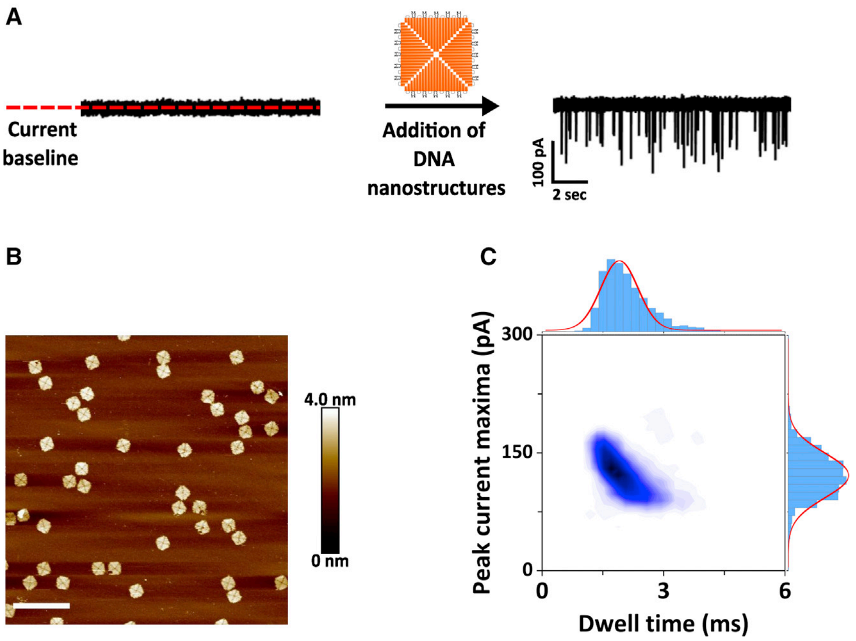

FIGURE 3 (*A*) Ion current trace before and after the addition of the DNA origami monomer. (*B*) AFM micrograph of a monomer DNA origami sample (400 nm scale bar). (*C*) Density scatter plot of the monomer DNA origami sample with the peak current maxima versus dwell time and their corresponding marginal histograms. The solid lines in the marginal histograms represent a Gaussian distribution fitted to the data. A total of $N = 2307$ translocation events were analyzed, and their corresponding peak current maxima and dwell time values extracted. To see this figure in color, go online.

Nanopore signal comparison of supramolecular DNA origami assemblies

We then analyzed the higher-order-assembly DNA origami samples using the same translocation conditions as for the monomer analysis. Fig. 4 *A* depicts the current versus time traces obtained for the monomer (*top panel*) and 2 × 2 (*bottom panel*) samples, where a new population of translocation events can be observed for the 2 × 2 sample.

While the monomer sample led to translocation events with peak current maxima of less than ~150 pA, events with significantly larger current amplitudes can be seen for the 2 × 2 sample—the current range within which these

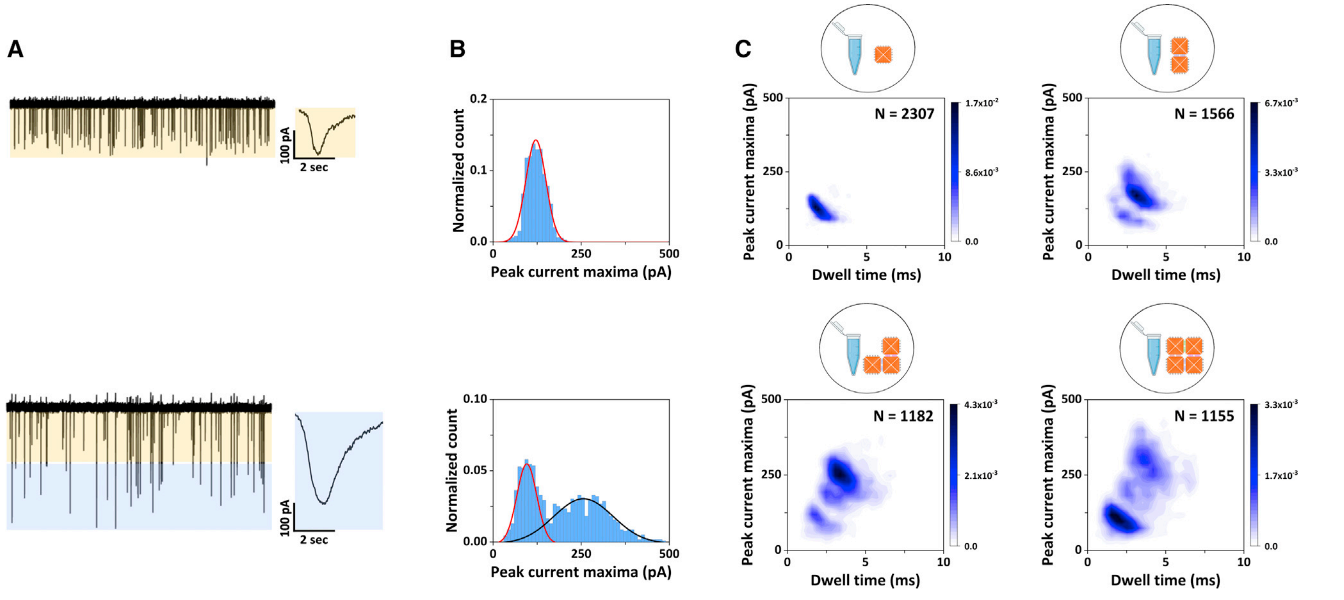

FIGURE 4 (*A*) Nanopore translocation current traces of the monomer (*top*) and the 2 × 2 (*bottom*) samples. The current and time scales for the two traces are identical. The orange shading indicates the current range <150 pA, and the blue shading indicates the current range >150 pA. The insets show a representative translocation event for each sample. The scale bars are the same for both insets. (*B*) Histograms of the current peak maxima for the monomer sample (*top*) and the 2 × 2 sample (*bottom*). The solid lines represent Gaussian fits to the data. (*C*) Density scatter plots of peak current maxima as a function of dwell time for the monomer sample (*top left*), dimer sample (*top right*), trimer sample (*bottom left*), and 2 × 2 sample (*bottom right*). The number of events analyzed, *N*, for each sample is given in each panel. To see this figure in color, go online.

new peaks fall is indicated by the blue shading. This observation suggested that our nanopore platform is able to discern the two "extreme" designs of the higher-order DNA origami assemblies introduced in Fig. 2 *A* using a fixed nanopore diameter. Similarly, the density scatter plots in Fig. 4 *C* show a clear difference in the clustering of the translocation events between the monomer (top left) and the 2 $\times$ 2 sample (bottom right). One of the clusters for the 2 $\times$ 2 sample is very similar to the single population observed in the monomer sample. However, the 2 $\times$ 2 sample shows an additional broader and less well-defined cluster centered at around 4 ms and 250 pA, which is also evidenced by the distributions plots in Fig. 4 *B*. The distributions were fitted with a single and a two-peak Gaussian distribution (shown as solid lines in the plot). While the monomer sample yielded an average peak current maximum of $\sim$123 pA (see also Fig. 3 *C*), the distribution corresponding to the more well-defined cluster in the 2 $\times$ 2 assembly sample density scatter plot, which is reminiscent of the one found for the monomer yielded an average peak current maximum of $\sim$100 pA and the broader less well-defined distribution an average peak current maximum of $\sim$260 pA. Despite the slight difference in average peak currents, we hypothesized that the more well-defined cluster of the 2 $\times$ 2 sample corresponds to monomers in the sample that were not successfully assembled into higher-order assemblies. We confirmed this hypothesis by spiking the 2 $\times$ 2 sample with increasing concentrations of the monomer. As can be seen in the Fig. S10, we observed a shift toward the monomer cluster when increasing the number of monomers spiked into the 2 $\times$ 2 sample. However, it is unlikely, given the relatively broad width of the distribution, that the less well-defined cluster represents only a single assembly state. This is further supported by both AFM imaging (Fig. S3) and gel electrophoresis , which suggest that the 2 $\times$ 2 sample did not only contain the 2 $\times$ 2 and monomer nanostructures but also other assembly intermediates. The range of characteristic translocation peaks of the 2 $\times$ 2 sample ion current are shown in Fig. S11. In order to deconvolute further the broad, less-well defined cluster of translocation events in the 2 $\times$ 2 sample, we analyzed all four DNA origami assembly samples, and the respective density scatter plots are shown in Fig. 4 *C*. All four DNA origami assembly samples (monomer, dimer, trimer, 2 $\times$ 2) were analyzed individually with the nanopore platform using the same translocation conditions. The density scatter plots allowed us to identify different clusters for each sample, and for all higher-order assembly samples (Fig. 4 *C*), we noticed a consistent presence of the monomer cluster. This is not unexpected—the higher-order assembly processes have finite yields, and a certain percentage of unassembled monomers are expected to remain. Furthermore, the higher-order assembly is reversible, and while the assembled construct is expected to be energetically favorable, dissociation of higher-order assemblies occurs over time. The translocation peak characteristics of each DNA origami sample showed differences in terms of their peak current maxima and dwell time distributions (Fig. S9). The dimer sample yielded one additional well-defined cluster of peaks, with slightly higher peak current maxima and dwell time averages, consistent with what is expected for the translocation of assembled dimers. However, the situation is much less clear for the trimer sample—similar to the 2 $\times$ 2 sample—where in addition to the well-defined cluster originating from the monomer peak, an additional number of much less-well defined clusters are observed. While the clustering is qualitatively different between the four samples, the density scatter plots cannot provide quantitative details about the assembly intermediates.

DNA origami assembly yield analysis

Finally, in addition to distinguishing different assembly intermediates within a mixed sample, we also aimed to go beyond the standard analysis of DNA origami nanostructure assembly (AFM and gel electrophoresis) and quantify the percentage of each DNA nanostructure present in the higher-order assembly samples. While the peak current maxima versus dwell time density scatter plots highlighted significant differences between samples, to extract more detail from the translocation information, we investigated an additional nanopore translocation discriminant. The observed translocation peaks are conductive and therefore imply that an increased amount of charge is passing through the nanopore during the translocation event compared with the baseline while no DNA nanostructures pass through. We define the equivalent charge surplus (ECS) of each translocation event as the area of the conductive translocation peak (Fig. 5 *A*) (37).

The ion current signature discriminants used above (peak current maximum and translocation dwell time) are likely dependent on the shape and orientation of the nanostructure during translocation through the nanopore. In contrast, the overall charge is expected to remain conserved for the same higher-order assembly state of DNA origami and can be expected to scale linearly with the size of the higher-order assemblies made from identical DNA origami (monomers). This is confirmed in Fig. 5 *B*, where the average ECS values of the DNA origami designs are plotted versus their surface area. The red line represents a linear fit to the data. Fig. 5 *C* depicts the ECS distributions for each higher-order DNA origami sample analyzed with the nanopore platform. In contrast to the distributions of the peak current maxima (Fig. 4 *B*), even for the most complex sample (2 $\times$ 2 sample), four clearly discernible peaks can be seen in the distribution, which suggests that each peak corresponds to either the monomer building block, the fully assembled nanostructure, or a particular higher-order assembly intermediate. To allocate the different distribution peaks to a particular nanostructure, we used the information

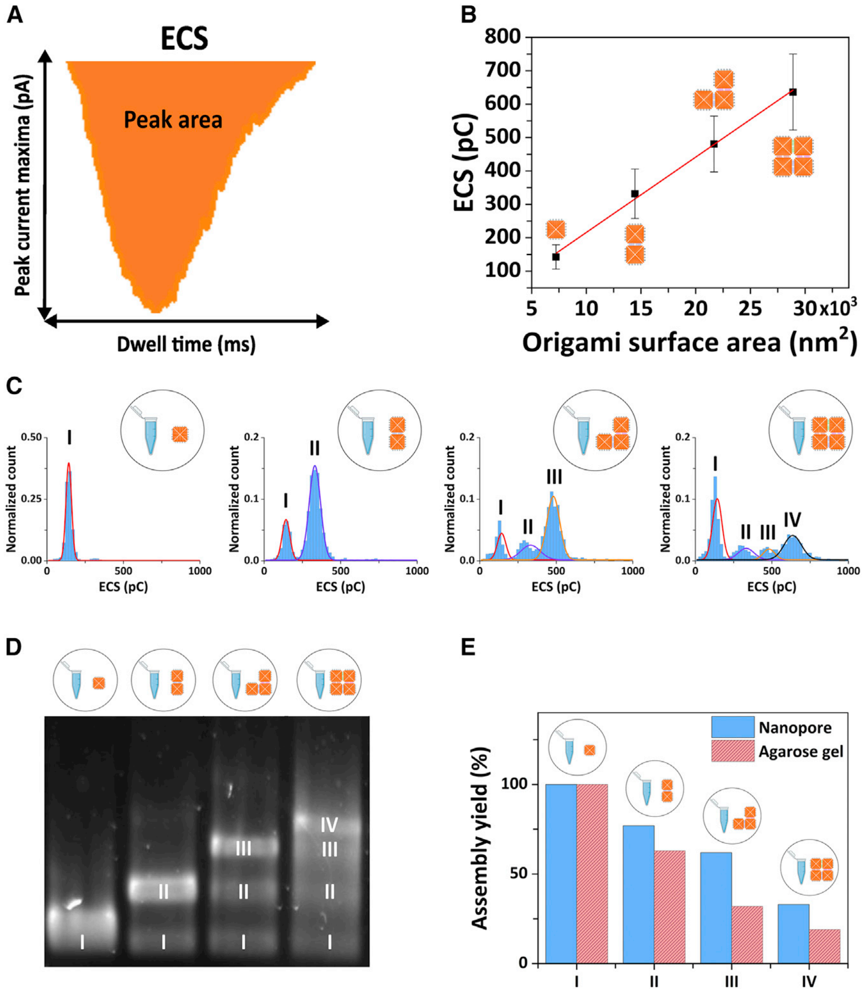

FIGURE 5 (*A*) Schematic representation of the concept of equivalent charge surplus (ECS), which is obtained from calculating the area of the translocation peaks. (*B*) ECS as a function of the DNA origami surface area for the four DNA nanostructure assembled (the red line represents a linear fit to the data). The error bars represent the width of the Gaussian fits displayed in (*C*). (*C*) ECS histograms of the DNA origami samples; from left to right: monomer sample, dimer sample, trimer sample, and 2 $\times$ 2 sample. The distributions were fitted with single or multi-peak Gaussian distributions. The notation I-IV marks the peaks corresponding to each of the DNA nanostructures in the respective sample (e.g., the dimer sample shows two peaks in the ECS distribution, attributed to the presence of the monomer (*I*) and dimer DNA origami (*II*) in the sample). (*D*) Agarose electrophoresis gel of the DNA origami samples; from left to right: monomer samples, dimer sample, trimer sample, and 2 $\times$ 2 sample. The I-IV notation of the gel bands marks the presence of the nanostructure component in the respective sample, similar to the notation used for the ECS distribution in (*C*) of the figure. (*E*) Bar chart comparison of the assembly yield of each DNA nanostructure (*blue bar*: nanopore data, *red bar*: gel electrophoresis data). To see this figure in color, go online.

obtained from less complex assemblies in the analysis of the more complex assemblies. Clearly, the monomer sample is expected to contain predominantly monomers and at most a negligible amount of non-specifically assembled higher-order structures. This is reflected in the very prominent peak centered around an ECS of $\sim$150 pC (Fig. 5 *C*, *leftmost panel*). The solid line represents a Gaussian fit to the distribution, which yielded an average ECS of 143 pC. The barely noticeable contribution to the distribution at around 300 pC may indicate a negligible presence of non-specific assemblies, but the contribution is difficult to quantify and is significantly smaller. Therefore, we allocate the ECS peak centered at 143 pC (labeled I) to the monomer. The ECS distribution of the dimer sample shows two peaks (Fig. 5 *C*, *second panel*). In this assembly sample, we expect the monomer and, of course, the dimer to be present. Therefore, we fitted the distribution with a two-peak Gaussian while keeping the first peak fixed at 143 pC, the position obtained from the monomer sample (Fig. 5 *C*, *leftmost panel*). The fits are represented by solid lines in the figure; the red line represents the fixed monomer peak (I) and the purple line the additional peak (II), which yielded an average ECS of 332 pC, and which we attribute to the presence of the dimer. To confirm that peak II indeed represents the dimer, we sliced the dimer sample events according to which Gaussian peak of the ECS distribution they belong and generated the associated peak current maxima versus dwell time density scatter plots (Fig. S12). As shown in the second row of Fig. S12, the monomer component in Fig. 4 *C* (*top left*) is not present in the density scatter plot (Fig. S12 *C*), supporting the viability of this ECS clustering to mark the different DNA nanostructure components present in the assembly samples. We then applied the same approach for the trimer and the 2 $\times$ 2 sample, resulting in an ECS distribution peak at 481 pC (III), which represents the trimer, and an ECS distribution peak at 636 pC (IV), which represents the 2 $\times$ 2 component. We note a slight discrepancy between the fixed monomer and dimer peaks in the ECS distributions compared with the observed distribution for both the trimer and 2 $\times$ 2. This may have resulted from small variations in pore size of the nanopipettes used, as each dataset was obtained with different nanopipettes. However, the deviations

are small and do not impact on our ability to distinguish different populations. The equivalent data slicing as for the dimer sample was carried out, and, like the dimer samples, the fully assembled DNA nanostructures yielded well-defined isolated clusters in the respective density scatter plots (Fig. S12 *C*). While all current traces were recordings of 3 min, we carried out the same analysis on a shorter trace (1 min) with correspondingly fewer translocation events, and we show in Fig. S13 that the results remain consistent for shorter current traces (1 versus 3 min recorded trace).

The areas of the Gaussian fits to the ECS distribution peaks allow us to associate individual translocation event to a distinct assembly state (monomer building block, fully assembled DNA nanostructure, or assembly intermediate) and thus to estimate the percentage of each higher-order DNA nanostructure present in the samples and compute an assembly yield for each DNA construct. Fig. 5 *E* (*blue bars*) shows the yield for forming the desired end product for each higher-order assembly sample, i.e., the percentage of monomer (I), dimer (II), trimer (III), and 2 × 2 (IV) in the monomer, dimer, trimer, and 2 × 2 samples, respectively. As expected from the histogram in Fig. 5 *C*, the yield for the monomer is 100%, while yields for the higher-order assemblies decrease with the increase in assembly size (all numerical yield values are supplied in Table S1). To further investigate the impact of the intrinsic variability of the nanopipettes' size for the quantification of the assembly yield, we carried out a number of translocation measurements of the same DNA origami sample using different but nominally identical nanopipettes. In Fig. S14, we show the ECS distribution based on the recordings of five nanopipettes and quantify the yield corresponding to the dimer DNA nanostructure for each repeat, showing a standard deviation in the calculated yield of 2% for 5 independent measurements (Table S2).

Assembly yield comparison with gel electrophoresis

Traditionally, agarose gel electrophoresis is employed to estimate assembly yields. An electrophoresis gel containing lanes for each higher-order assembly is shown in Fig. 5 *D*, and the assembly yields were determined from the agarose gel by densitometry and taking into account the size of the structure. The results are shown as red bars in Fig. 5 *E*, and the numerical values are shown in Table S1. Overall, the yields obtained with gel electrophoresis show the same trend as the ones obtained from nanopore measurements. However, there are some notable differences. The agarose gel indicated a larger percentage of monomers in the higher-order assemblies for the trimer and 2 × 2 sample compared with the nanopore measurements (Table S1). Our approach is comparable to gel electrophoresis in terms of costs and ease of use but with the added advantage of single-molecule sensitivity and the ability to obtain quantitative results within minutes (Fig. S13). Another often employed means of assessing the yield of DNA origami structures is AFM. Our AFM micrographs (Fig. S3) confirmed the heterogeneous character of the higher-order DNA origami samples. AFM is extremely well suited to study small numbers of DNA origami at very high detail so a good understanding of their structures and fine detail of the folding can be obtained. Previous studies demonstrated the use of AFM imaging to obtain quantification yields of folded DNA nanostructures based on the molecule count from AFM scans in solution (35,43,44). However, their use for quantification of the assembly yield is questionable in this case. AFM scans depend heavily on the mica surface preparation that is required for imaging, and this is likely to lead to an under-representation of smaller DNA constructs owing to the higher sedimentation rate of the larger DNA origami structures onto the mica substrate. This was confirmed here, where we observed an under-representation of monomers in the higher-order assembly samples (trimer and 2 × 2 samples) compared with the nanopore measurements.

Compared with the standard analysis methods of AFM and agarose gel electrophoresis, the nanopore measurements offer several advantages. Our nanopore method enables a label-free analysis of the DNA origami samples within a few minutes with single-molecule resolution at statistically relevant numbers and no lengthy sample preparations or use of expensive reagents. Another advantage of the nanopore approach is its single-molecule analysis, which can potentially detect minute concentrations and reveal the presence of DNA constructs that have formed with very low yields. In Fig. S8 *F*, we demonstrate nanopore detection of DNA origami nanostructures down to the 50 pM concentrations.

In addition to utilizing nanopore measurements for the determination of assembly yields of DNA origami, the approach enables a range of other applications. For example, the ability to differentiate between assembly states enables the probing of the association/dissociation of higher-order DNA constructs and shed light on their stability in different assembly configurations. Furthermore, the analysis is in real time and non-destructive, i.e., the DNA origami nanostructures could be collected in the bath after translocation and reused. This opens up the possibility for using the nanopore approach in label-free separation or purification, where, depending on the translocation peak characteristics, the DNA nanostructure can be steered (e.g., electrophoretically) into a collection or waste tube. Future developments of the nanopore measurement approach will include the parallelization of measurement using arrays of nanopores to increase throughput. Previous studies demonstrated the fabrication of solid-state nanopore arrays on silicon nitride or graphene membranes (45,46), which are manufactured using inherently scalable approaches. Similarly, Alawami et al. have shown the use of multiple glass nanopipettes embedded in

a PDMS devices (47). Furthermore, machine-learning approaches integrated with nanopore measurements can lead to a real-time classification of the DNA nanostructures. Examples of combining nanopore sensing with machine learning has been demonstrated for different analytes (48–50). As the field of DNA origami is expanding at a fast pace, more intricate structures in terms of design and functionality emerge, requiring the integration of additional nanopore features, like machine-learning algorithms, to provide accurate and robust identification and quantification of complex supramolecular structures.

CONCLUSIONS

In conclusion, we explored nanopore translocation as a single-molecule approach to probe the heterogeneous character of DNA origami assemblies. The large number of events that can be recorded (>1000 events) for each sample within minutes enables statistically relevant studies in a non-destructive and label-free way. We demonstrated the discrimination of various assembly states for higher-order DNA origami assemblies based on their equivalent charge surplus computed from the recorded ion current signals, which allowed the quantification of the assembly yields without any lengthy sample preparations and, importantly, enables a range of other applications where rapid single-molecule detection is required. Our work complements related approaches of using nanopore translocations characteristics to differentiate between DNA nanostructures with different geometries (51–53), further enabling the analysis of higher-order assemblies.

DATA AVAILABILITY

Data supporting this work can be accessed via the University of Leeds repository: https://doi.org/10.5518/1198.

SUPPORTING MATERIAL

Supporting material can be found online at https://doi.org/10.1016/j.bpj.2022.08.020.

AUTHOR CONTRIBUTIONS

S.C. performed the nanopore sensing, gel electrophoresis, AFM measurements, and analyzed the data. G.M. performed nanopore sensing experiments and analyzed the data. I.S. designed and formed the DNA nanostructures and performed AFM measurements. C.W. and P.A supervised the research and supported the data analysis. All authors wrote the manuscript.

ACKNOWLEDGMENTS

We thank Dr. Alexander Kulak for the help provided with scanning electron microscopy imaging of the nanopipettes. S.C. and P.A. acknowledge funding from the European Union's Horizon 2020 research and innovation 7 program under the Marie Skłodowska-Curie MSCA-ITN grant agreement no. 812398, through the single-entity nanoelectrochemistry, SENTINEL, project. I.S. was supported by funding from the European Research Council (ERC) under the project DYNAMIN, grant agreement no. 788968. G.M. acknowledges funding from a University of Leeds Scholarship. P.A and C.W. acknowledge funding from the Engineering and Physical Sciences Research Council UK (EPSRC) Healthcare Technologies for the grant EP/W004933/1, and C.W. acknowledges funding from the Medical Research Council (MRC) UK under the grant number MR/N029976/1. Schematics used in our figures were adapted from BioRender.com.

DECLARATION OF INTERESTS

The authors declare no competing interests.

REFERENCES

1. Seeman, N. C., and H. F. Sleiman. 2017. DNA nanotechnology. *Nat. Rev. Mater.* 3:17068. https://doi.org/10.1038/natrevmats.2017.68.
2. Dey, S., C. Fan, ..., P. Zhan. 2021. DNA origami. *Nat. Rev. Methods Primers.* 1:13. https://doi.org/10.1038/s43586-020-00009-8.
3. Chandrasekaran, A. R., N. Anderson, ..., X. Wang. 2016. Beyond The fold: emerging biological applications of DNA origami. *Chembiochem.* 17:1081–1089. https://doi.org/10.1002/cbic.201600038.
4. Engelhardt, F. A. S., F. Praetorius, ..., H. Dietz. 2019. Custom-size, functional, and durable DNA origami with design-specific scaffolds. *ACS Nano.* 13:5015–5027. https://doi.org/10.1021/acsnano.9b01025.
5. Zhang, Y., Q. Li, ..., L. Wang. 2020. Prescribing DNA origami patterns via scaffold decoration. *Small.* 16:e2000793. https://doi.org/10.1002/smll.202000793.
6. Tikhomirov, G., P. Petersen, and L. Qian. 2017. Fractal assembly of micrometre-scale DNA origami arrays with arbitrary patterns. *Nature.* 552:67–71. https://doi.org/10.1038/nature24655.
7. Wang, X., H. Jun, and M. Bathe. 2022. Programming 2D supramolecular assemblies with wireframe DNA origami. *J. Am. Chem. Soc.* 144:4403–4409. https://doi.org/10.1021/jacs.1c11332.
8. Gu, H., J. Chao, ..., N. C. Seeman. 2009. Dynamic patterning programmed by DNA tiles captured on a DNA origami substrate. *Nat. Nanotechnol.* 4:245–248. https://doi.org/10.1038/nnano.2009.5.
9. Huang, Y., M.-K. Nguyen, ..., A. Kuzyk. 2018. A DNA origami-based chiral plasmonic sensing device. *ACS Appl. Mater. Interfaces.* 10:44221–44225. https://doi.org/10.1021/acsami.8b19153.
10. Arroyo-Currás, N., M. Sadeia, ..., P. S. Lukeman. 2020. An electrochemical biosensor exploiting binding-induced changes in electron transfer of electrode-attached DNA origami to detect hundred nanometer-scale targets. *Nanoscale.* 12:13907–13911. https://doi.org/10.1039/D0NR00952K.
11. Selnihhin, D., S. M. Sparvath, ..., E. S. Andersen. 2018. Multifluorophore DNA origami beacon as a biosensing platform. *ACS Nano.* 12:5699–5708. https://doi.org/10.1021/acsnano.8b01510.
12. Raveendran, M., A. R. Leach, ..., P. Actis. 2020. Ribosome fingerprinting with a solid-state nanopore. *ACS Sens.* 5:3533–3539. https://doi.org/10.1021/acssensors.0c01642.
13. Raveendran, M., A. J. Lee, ..., P. Actis. 2020. Rational design of DNA nanostructures for single molecule biosensing. *Nat. Commun.* 11:4384. https://doi.org/10.1038/s41467-020-18132-1.
14. Zhang, Q., Q. Jiang, ..., Y. Du. 2014. DNA origami as an in vivo drug delivery vehicle for cancer therapy. *ACS Nano.* 8:6633–6643. https://doi.org/10.1021/nn502058j.
15. Ge, Z., L. Guo, ..., Q. Li. 2020. DNA origami-enabled engineering of ligand-drug conjugates for targeted drug delivery. *Small.* 16:e1904857. https://doi.org/10.1002/smll.201904857.

16. Lee, A. J., M. Endo, ..., C. Wälti. 2018. Direct single-molecule observation of mode and geometry of RecA-mediated homology search. *ACS Nano.* 12:272–278. https://doi.org/10.1021/acsnano.7b06208.

17. Lee, A. J., M. Endo, ..., C. Wälti. 2021. Micro-homology intermediates: RecA's transient sampling revealed at the single molecule level. *Nucleic Acids Res.* 49:1426–1435. https://doi.org/10.1093/nar/gkaa1258.

18. Platnich, C. M., F. J. Rizzuto, ..., H. F. Sleiman. 2020. Single-molecule methods in structural DNA nanotechnology. *Chem. Soc. Rev.* 49:4220–4233. https://doi.org/10.1039/C9CS00776H.

19. Xue, L., H. Yamazaki, ..., J. B. Edel. 2020. Solid-state nanopore sensors. *Nat. Rev. Mater.* 5:931–951. https://doi.org/10.1038/s41578-020-0229-6.

20. Alfaro, J. A., P. Bohländer, ..., C. Joo. 2021. The emerging landscape of single-molecule protein sequencing technologies. *Nat. Methods.* 18:604–617. https://doi.org/10.1038/s41592-021-01143-1.

21. Shi, W., A. K. Friedman, and L. A. Baker. 2017. Nanopore sensing. *Anal. Chem.* 89:157–188. https://doi.org/10.1021/acs.analchem.6b04260.

22. Houghtaling, J., C. Ying, ..., M. Mayer. 2019. Estimation of shape, volume, and dipole moment of individual proteins freely transiting a synthetic nanopore. *ACS Nano.* 13:5231–5242. https://doi.org/10.1021/acsnano.8b09555.

23. Bacri, L., A. G. Oukhaled, ..., L. Auvray. 2011. Dynamics of colloids in single solid-state nanopores. *J. Phys. Chem. B.* 115:2890–2898. https://doi.org/10.1021/jp200326w.

24. Karmi, A., H. Dachlika, ..., D. Porath. 2021. Detection of Au nanoparticles using peptide-modified Si3N4 nanopores. *ACS Appl. Nano Mater.* 4:1000–1008. https://doi.org/10.1021/acsanm.0c02126.

25. Arima, A., I. H. Harlisa, ..., T. Kawai. 2018. Identifying single viruses using biorecognition solid-state nanopores. *J. Am. Chem. Soc.* 140:16834–16841. https://doi.org/10.1021/jacs.8b10854.

26. Yu, R.-J., S.-M. Lu, ..., Y.-T. Long. 2019. Single molecule sensing of amyloid-β aggregation by confined glass nanopores. *Chem. Sci.* 10:10728–10732. https://doi.org/10.1039/C9SC03260F.

27. Bell, N. A. W., C. R. Engst, ..., U. F. Keyser. 2012. DNA origami nanopores. *Nano Lett.* 12:512–517. https://doi.org/10.1021/nl204098n.

28. Wang, V., N. Ermann, and U. F. Keyser. 2019. Current enhancement in solid-state nanopores depends on three-dimensional DNA structure. *Nano Lett.* 19:5661–5666. https://doi.org/10.1021/acs.nanolett.9b02219.

29. Plesa, C., N. van Loo, ..., C. Dekker. 2015. Velocity of DNA during translocation through a solid-state nanopore. *Nano Lett.* 15:732–737. https://doi.org/10.1021/nl504375c.

30. Plesa, C., A. N. Ananth, ..., C. Dekker. 2014. Ionic permeability and mechanical properties of DNA origami nanoplates on solid-state nanopores. *ACS Nano.* 8:35–43. https://doi.org/10.1021/nn405045x.

31. Raveendran, M., A. J. Lee, ..., P. Actis. 2018. Analysis of 2D DNA origami with nanopipettes. *Chemelectrochem.* 5:3014–3020. https://doi.org/10.1002/celc.201800732.

32. Chau, C. C., S. E. Radford, ..., P. Actis. 2020. Macromolecular crowding enhances the detection of DNA and proteins by a solid-state nanopore. *Nano Lett.* 20:5553–5561. https://doi.org/10.1021/acs.nanolett.0c02246.

33. Beamish, E., V. Tabard-Cossa, and M. Godin. 2019. Programmable DNA nanoswitch sensing with solid-state nanopores. *ACS Sens.* 4:2458–2464. https://doi.org/10.1021/acssensors.9b01053.

34. Chen, K., J. Zhu, F. Bošković, and U. F. Keyser. 2020. Nanopore-based DNA hard drives for rewritable and secure data storage. *Nano Lett.* 20:3754–3760. https://doi.org/10.1021/acs.nanolett.0c00755.

35. Tikhomirov, G., P. Petersen, and L. Qian. 2017. Programmable disorder in random DNA tilings. *Nat. Nanotechnol.* 12:251–259. https://doi.org/10.1038/nnano.2016.256.

36. Douglas, S. M., A. H. Marblestone, ..., W. M. Shih. 2009. Rapid prototyping of 3D DNA-origami shapes with caDNAno. *Nucleic Acids Res.* 37:5001–5006. https://doi.org/10.1093/nar/gkp436.

37. Plesa, C., and C. Dekker. 2015. Data analysis methods for solid-state nanopores. *Nanotechnology.* 26:084003. https://doi.org/10.1088/0957-4484/26/8/084003.

38. Chau, C. C., F. Marcuccio, ..., P. Actis. 2021. Cooperative electrolyte-PEG interactions drive the signal amplification in a solid-state nanopore. Preprint at bioRxiv. https://doi.org/10.1101/2021.11.01.466478.

39. Ren, R., M. Sun, P. Goel, ..., J. B. Edel. 2021. Single-molecule binding assay using nanopores and dimeric NP conjugates. *Adv. Mater.* 33:2103067. https://doi.org/10.1002/adma.202103067.

40. Wanunu, M., J. Sutin, ..., A. Meller. 2008. DNA translocation governed by interactions with solid-state nanopores. *Biophys. J.* 95:4716–4725. https://doi.org/10.1529/biophysj.108.140475.

41. Li, J., and D. S. Talaga. 2010. The distribution of DNA translocation times in solid-state nanopores. *J. Phys. Condens. Matter.* 22:454129. https://doi.org/10.1088/0953-8984/22/45/454129.

42. Zhu, L., Z. Zhang, and Q. Liu. 2020. Deformation-mediated translocation of DNA origami nanoplates through a narrow solid-state nanopore. *Anal. Chem.* 92:13238–13245. https://doi.org/10.1021/acs.analchem.0c02396.

43. Zenk, J., C. Tuntivate, and R. Schulman. 2016. Kinetics and thermodynamics of watson–crick base pairing driven DNA origami dimerization. *J. Am. Chem. Soc.* 138:3346–3354. https://doi.org/10.1021/jacs.5b10502.

44. Dunn, K. E., F. Dannenberg, ..., J. Bath. 2015. Guiding the folding pathway of DNA origami. *Nature.* 525:82–86. https://doi.org/10.1038/nature14860.

45. Kim, M. J., M. Wanunu, ..., A. Meller. 2006. Rapid fabrication of uniformly sized nanopores and nanopore arrays for parallel DNA analysis. *Adv. Mater.* 18:3149–3153. https://doi.org/10.1002/adma.200601191.

46. Verschueren, D. V., W. Yang, and C. Dekker. 2018. Lithography-based fabrication of nanopore arrays in freestanding SiN and graphene membranes. *Nanotechnology.* 29:145302. https://doi.org/10.1088/1361-6528/aaabce.

47. Alawami, M. F., F. Bošković, ..., U. F. Keyser. 2022. Lifetime of glass nanopores in a PDMS chip for single-molecule sensing. *iScience.* 25:104191. https://doi.org/10.1016/j.isci.2022.104191.

48. Meyer, N., J. M. Janot, ..., S. Balme. 2020. Machine learning to improve the sensing of biomolecules by conical track-etched nanopore. *Biosensors.* 10:140. https://doi.org/10.3390/bios10100140.

49. Taniguchi, M., H. Takei, ..., N. Naono. 2022. Sensing the performance of artificially intelligent nanopores developed by integrating solid-state nanopores with machine learning methods. *J. Phys. Chem. C.* 126:12197–12209. https://doi.org/10.1021/acs.jpcc.2c02674.

50. Taniguchi, M., S. Minami, ..., K. Tomono. 2021. Combining machine learning and nanopore construction creates an artificial intelligence nanopore for coronavirus detection. *Nat. Commun.* 12:3726. https://doi.org/10.1038/s41467-021-24001-2.

51. Xie, Z., S. Liu, and Y. Zhai. 2022. Study on the self-assembly and signal amplification ability of nucleic acid nanostructure with the nanopipette. *J. Electroanal. Chem.* 914:116307. https://doi.org/10.1016/j.jelechem.2022.116307.

52. Yang, J., N. Zhao, ..., C. Zhang. 2021. Structure-flexible DNA origami translocation through a solid-state nanopore. *RSC Adv.* 11:23471–23476. https://doi.org/10.1039/D1RA04267J.

53. Alibakhshi, M. A., J. R. Halman, ..., M. Wanunu. 2017. Picomolar fingerprinting of nucleic acid nanoparticles using solid-state nanopores. *ACS Nano.* 11:9701–9710. https://doi.org/10.1021/acsnano.7b04923.

Biophysical Journal, Volume *121*

Supplemental information

Nanopore fingerprinting of supramolecular DNA nanostructures

Samuel Confederat, Ilaria Sandei, Gayathri Mohanan, Christoph Wälti, and Paolo Actis

Supporting Material: Nanopore Fingerprinting of Supramolecular DNA Origami Nanostructures

Samuel Confederat[1,2], Ilaria Sandei[3], Gayathri Mohanan[1,2], Christoph Wälti[1,2], Paolo Actis[1,2]

[1] School of Electronic and Electrical Engineering and Pollard Institute, University of Leeds, Leeds LS2 9JT, UK

[2] Bragg Centre for Materials Research, Leeds, LS2 9JT, UK

[3] School of Chemistry, University of Leeds, Leeds LS2 9JT, UK

KEYWORDS: nanopore, nanopipette, DNA origami, single molecule, AFM, bionanotechnology, DNA nanotechnology

* Corresponding authors: c.walti@leeds.ac.uk; p.actis@leeds.ac.uk

S1: Nanopore characterization 2

S2: Agarose gel electrophoresis analysis of DNA nanostructures 3

S3: AFM analysis of DNA origami samples 3

S4: Nanopore detection enhancement in PEG electrolyte bath 4

S5: Nanopore measurement stability 4

S6: Translocation events detection from nanopore current traces 4

S7: Translocation controls DNA nanostructures 5

S8: Translocation event characteristics DNA nanostructures 6

S9: Translocation events comparison for different DNA origami samples 7

S10: Monomer spiking 8

S11: Representative translocation peaks 8

S12: DNA nanostructures cluster isolation based on ECS 9

S13: Ratio of DNA nanostructures at different number of translocation events 9

S14: Ratio of DNA nanostructures nanopore *vs* gel electrophoresis 10

S15: DNA nanostructure yield based on nanopipette repeats 10

S16: DNA nanostructures design 11

References 11

S1: Nanopore characterization

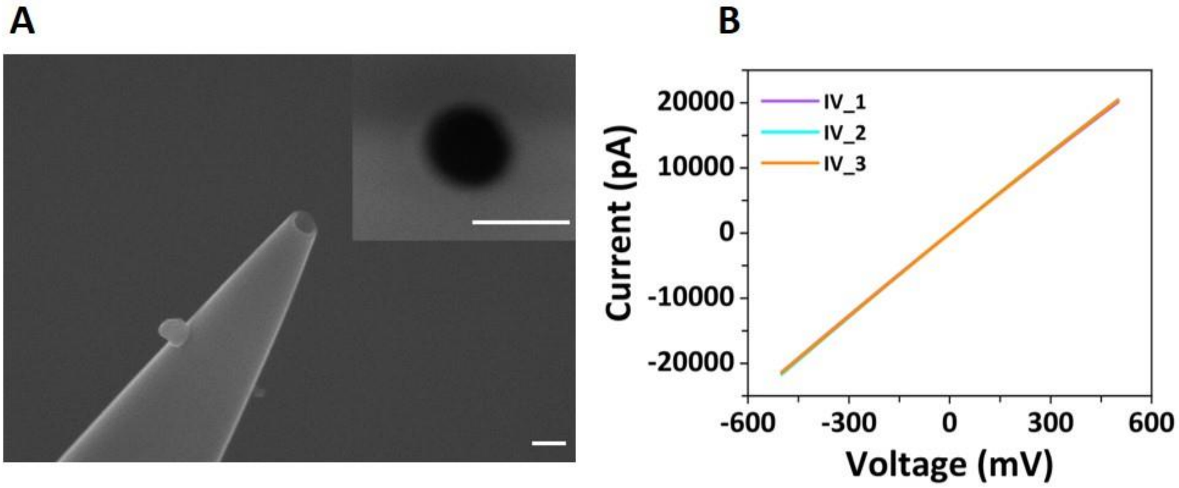

Figure S1: (A) SEM micrograph of a nanopipette used for translocation experiments, with a top view inset of the pore (200 nm scale bars). The diameter of the pore used in this study was approximately 160 nm. **(B)** Current-voltage curves (IV) of three nanopipettes recorded in 0.1 M KCl.

S2: Agarose gel electrophoresis analysis of DNA nanostructures

Quantification of the relative amounts of monomers, various assembly intermediates, and final products for each DNA origami sample was carried out by measuring the corresponding bands' intensity with ImageJ and normalize by the size of the higher-order assemblies to account for the amount of double stranded DNA stained per macromolecule. As shown in Figure S2, the gel electrophoresis analysis confirmed the presence of multiple DNA nanostructures in the higher-order assembly samples, *i.e.* the dimer sample contains the monomer nanostructures as well. The trimer sample contains monomer and dimer nanostructures. The 2x2 samples contains monomer, dimer, and trimer nanostructures.

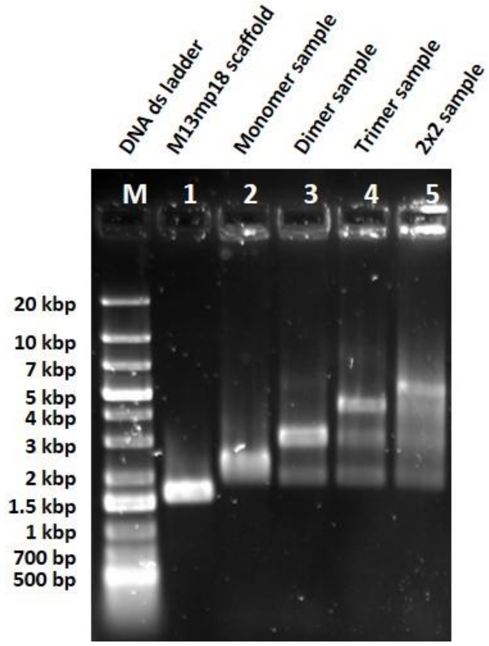

Figure S2: Agarose gel electrophoresis of DNA origami samples used in this study. Lane M: GeneRuler 1kb dsDNA ladder; Lane 1: M13mp18 circular ssDNA; Lane 2: monomer DNA origami sample; Lane 3: dimer DNA origami sample; Lane 4: trimer DNA origami sample; Lane 5: 2x2 DNA origami sample. Note, Figure 6D is a subset of this figure.

S3: AFM analysis of DNA origami samples

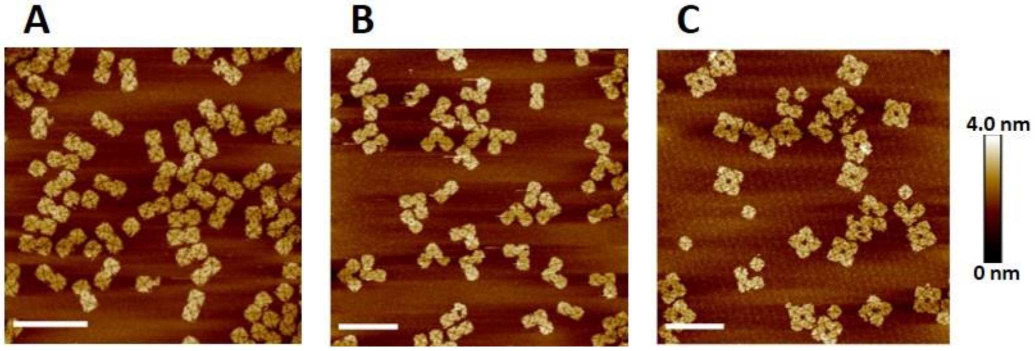

Figure S3: AFM micrographs of the higher-order assembly DNA origami samples: dimer **(A)**, trimer **(B)**, and 2x2 **(C)** using a 2 µm x 2 µm scan size (400 nm scale bars).

S4: Nanopore detection enhancement in PEG electrolyte bath

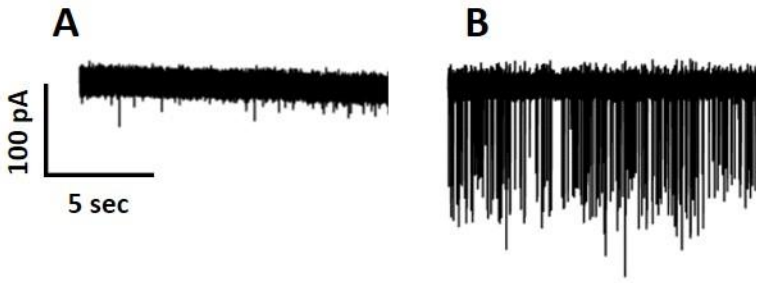

Figure S4: Ion current traces for the monomer DNA origami sample recorded in 0.1 M KCl electrolyte bath **(A)** and 50% (w/v) PEG 35k 0.1 M KCl bath **(B)**. Current traces were recorded at -300mV using the same nanopipette filled with monomer DNA nanostructures. The current and time scales are identical for both plots.

S5: Nanopore measurement stability

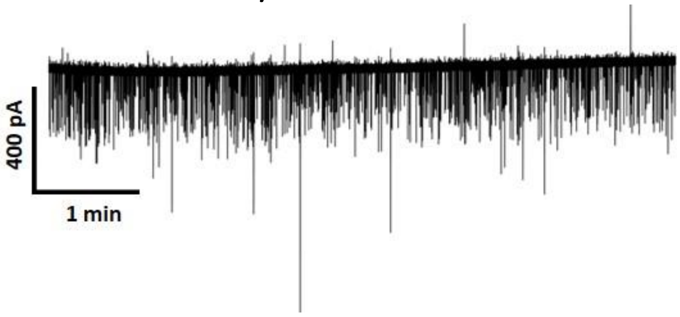

Figure S5: Ion Current trace of the 2x2 DNA origami sample recorded for 6 minutes under an applied voltage of -300mV.

S6: Translocation events detection from nanopore current traces

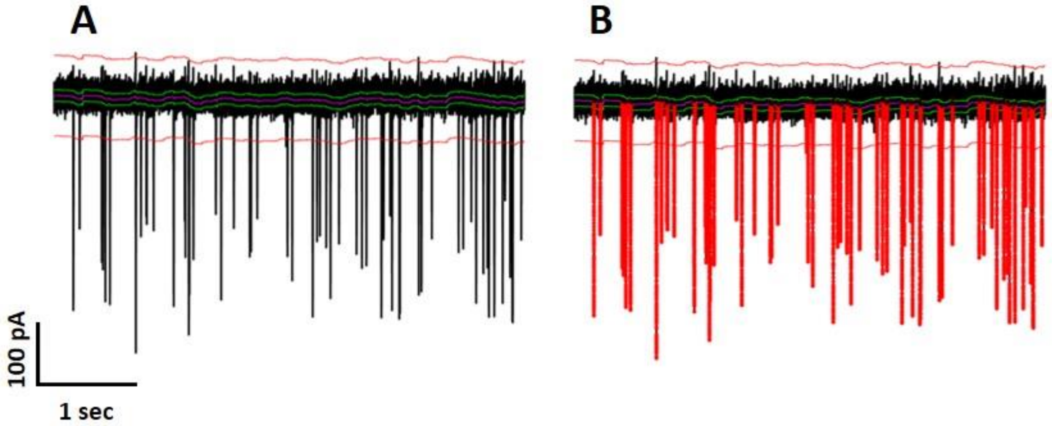

Figure S6: **(A)** Representative ion current trace with the 7σ threshold indicated by the red line. **(B)** Same trace as in A with the perturbations in the ion current identified as DNA origami translocation events by the Transanalyser Matlab script (1) according to the 7σ threshold highlighted in red.

S7: Translocation controls DNA nanostructures

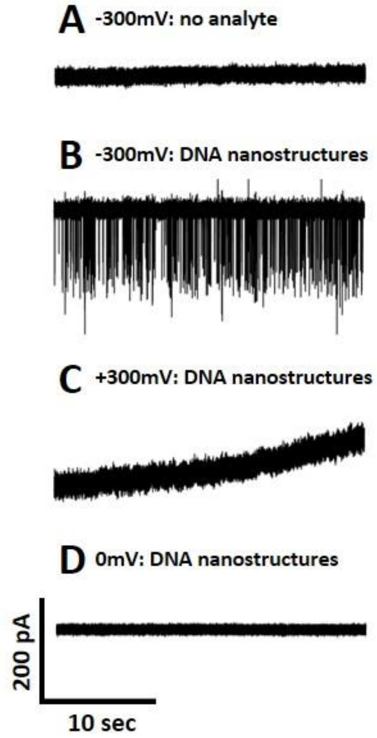

Figure S7: Control ion currents traces. **(A)** translocation at -300 mV when no analyte is added inside the nanopipette; **(B)** translocation at -300 mV when monomer DNA origami sample is added inside the nanopipette; **(C)** translocation at +300 mV when monomer DNA origami sample is added inside the nanopipette; **(D)** translocation at 0 mV when monomer DNA origami sample is added inside the nanopipette. The current and timescales are the same for all graphs.

S8: Translocation event characteristics DNA nanostructures

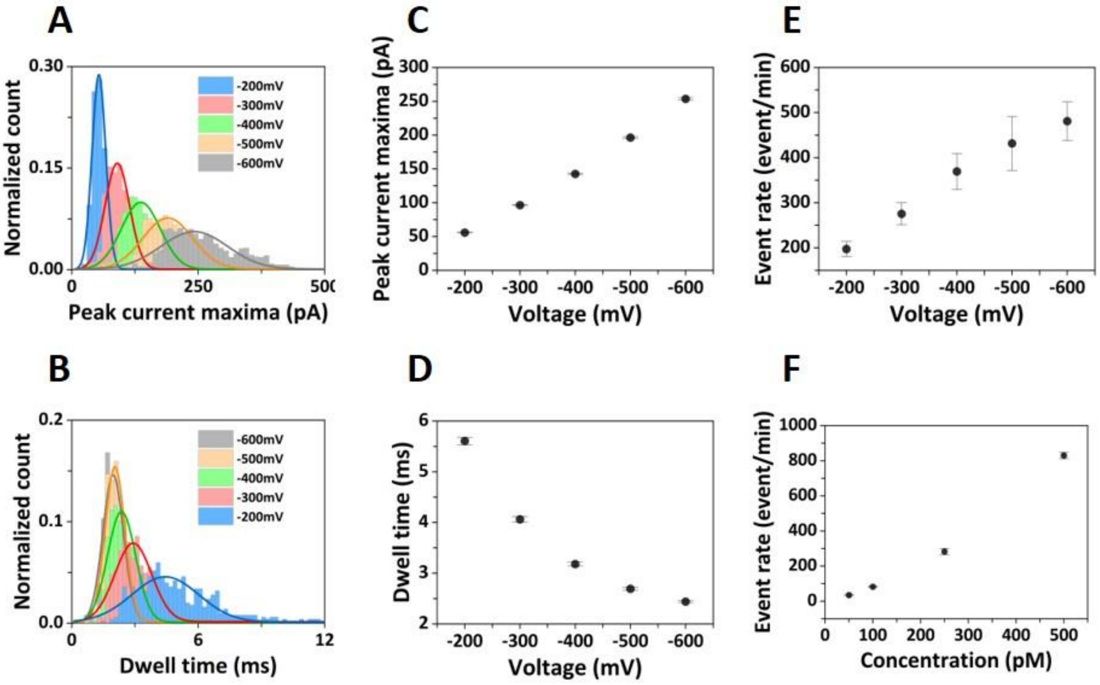

Figure S8: Histograms of the current peak maxima **(A)** and the dwell time **(B)** distributions for increasing absolute values of applied voltages *V* = -200 mV, -300 mV, -400 mV, -500 mV, and -600 mV. The solid lines represent Gaussian fits to the distributions. Average peak current maxima **(C)** and dwell time **(D)** at *V* = -200 mV, -300 mV, -400 mV, -500 mV, and -600 mV. Error bars show the standard deviation from three independent recordings. Event rate of the DNA nanostructures translocation as a function of *V* **(E)** and sample concentration (50 pM, 100 pM, 250 pM, and 500 pM) **(F)**. Error bars show the standard deviation from three independent recordings. Monomer DNA origami sample (250 pM for panel A-E) was used for these translocation experiments, and each translocation condition was repeated independently three times.

S9: Translocation events comparison for different DNA origami samples

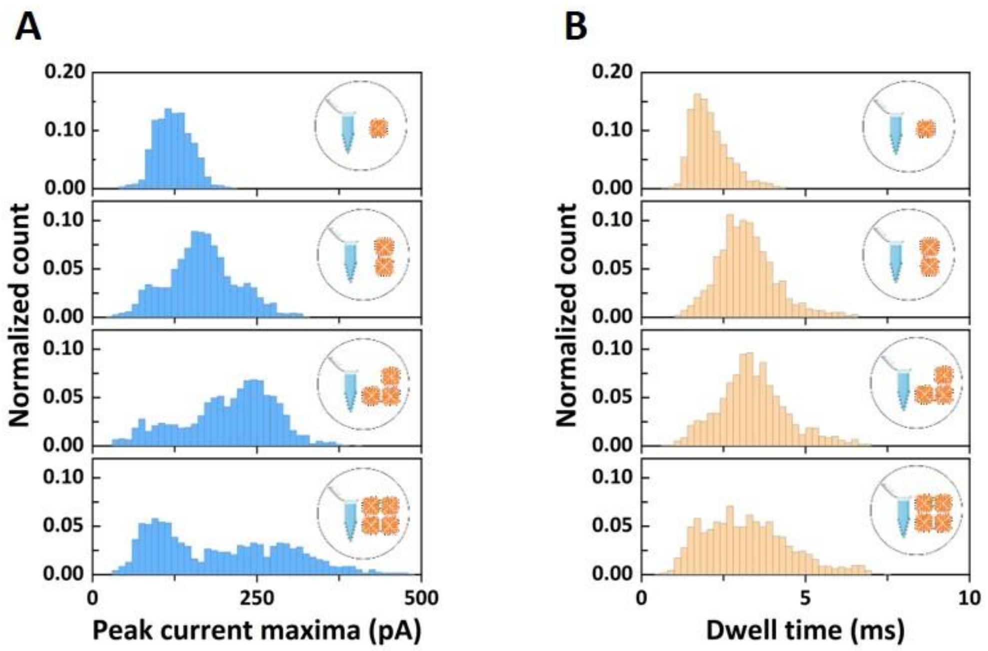

Figure S9: Histograms for peak current maxima **(A)** and dwell time **(B)** distributions for each DNA origami sample investigated. From top to bottom: monomer, dimer, trimer, and 2x2 samples. Translocation events were recorded at -300 mV.

S10: Monomer spiking

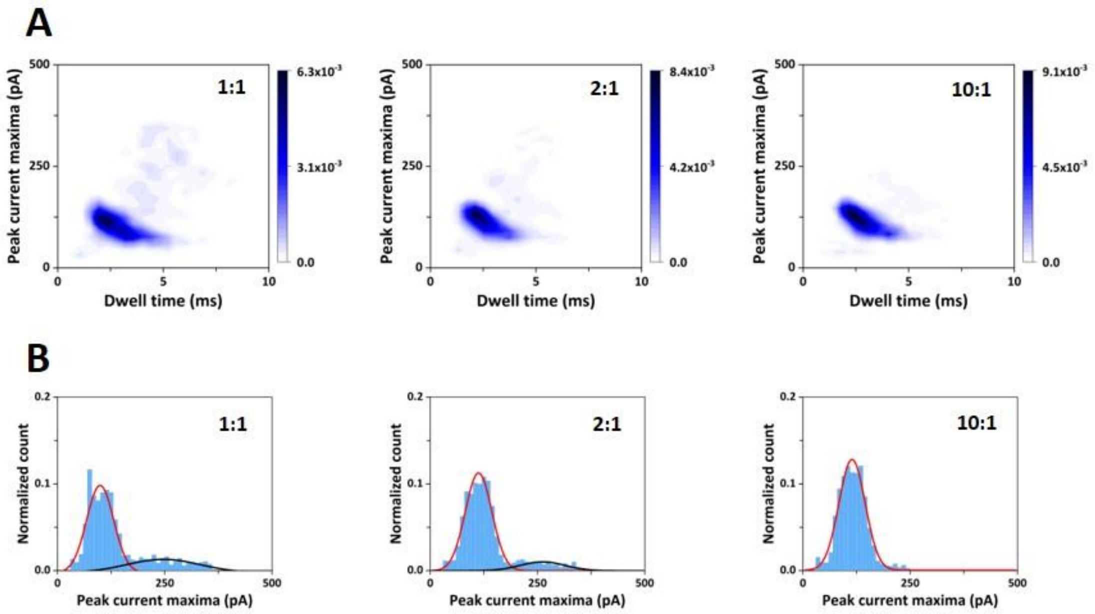

Figure S10: **(A)** Density scatter plots of the peak current maxima as a function of dwell time for 2x2 sample spiked with monomer sample. **(B)** Histograms of the current peak maxima distributions for the 2x2 sample spiked with monomer sample with multi-peak Gaussian fits represented by the solid lines. The notation 1:1, 2:1, and 10:1 for the left-to-right graphs in each panel indicates the molar ratio of monomer sample spiked into the 2x2 sample.

S11: Representative translocation peaks

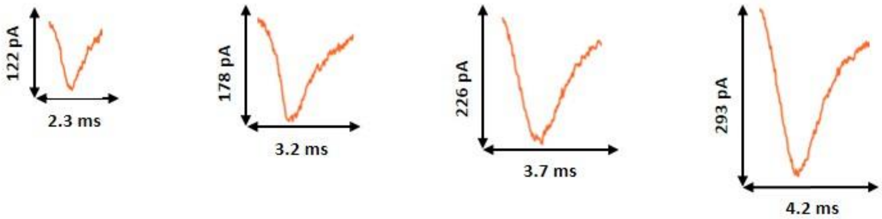

Figure S11: Representative translocation peaks characteristic of the heterogeneous 2x2 sample with their corresponding dwell time and peak current maxima values.

S12: DNA nanostructures cluster isolation based on ECS

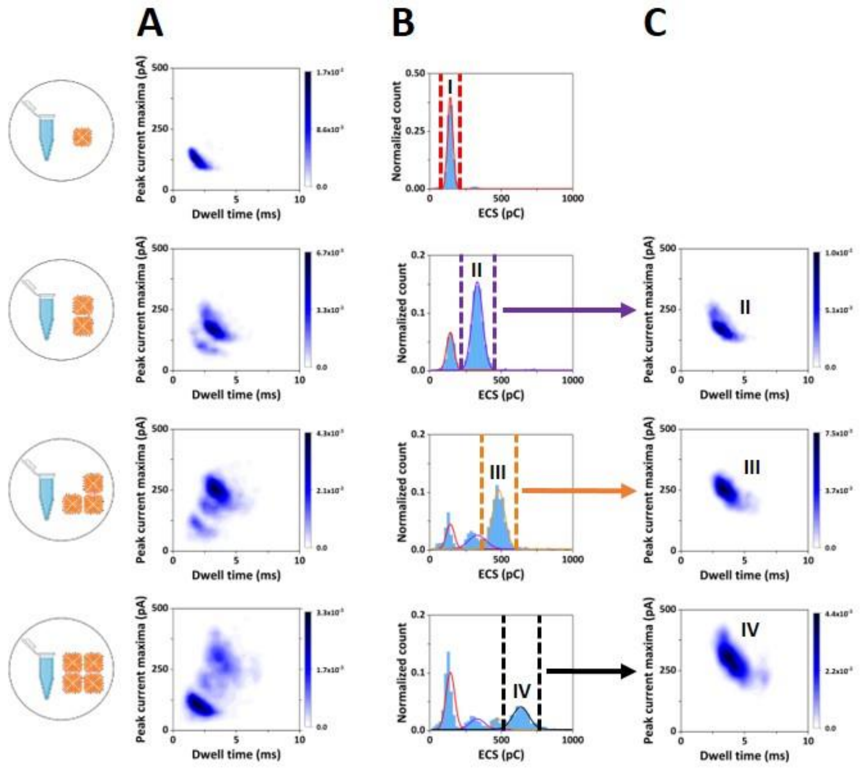

Figure S12: (A) Density scatter plots of the current peak maxima as a function of dwell time for (top to bottom): monomer sample, dimer sample, trimer sample, and 2x2 sample. **(B)** ECS histograms for the DNA origami samples presented in panel A. Marked Gaussian fitted peaks correspond to the DNA nanostructures of interest in each sample. **(C)** The corresponding sliced cluster depicted in a density scatter plot using only the translocation events sliced according to the marked Gaussian fitted peaks shown in Panel B. We note that the intensity scales are normalised to the number of translocation events shown, and therefore are different compared to panel A.

S13: Ratio of DNA nanostructures at different number of translocation events

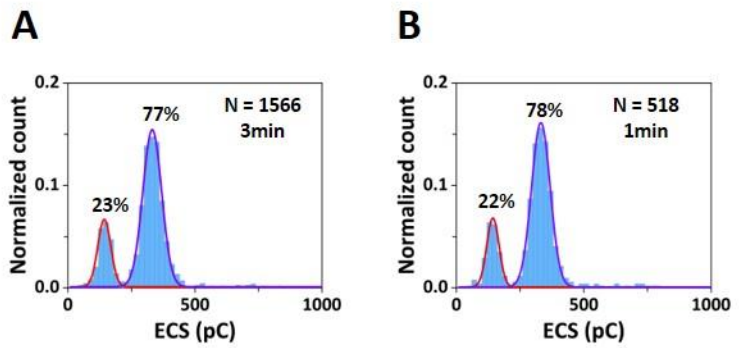

Figure S13: ECS histograms for dimer sample based on a 3-minute translocation recording **(A)** and 1-minute translocation recording **(B)**. Solid lines are Gaussian fits to the distributions.

S14: Ratio of DNA nanostructures nanopore *vs* gel electrophoresis

Table S1: Percentage of DNA nanostructures determined for each DNA origami sample analyzed. The notation I-IV refers to the DNA origami nanostructures present in the sample analyzed: I - monomer nanostructure, II - dimer nanostructure, III - trimer nanostructure, and IV - 2x2 nanostructures. The dash indicates that the component was not detected and no percentage was computed.

DNA origami sample	Ratio DNA nanostructure nanopore analysis (%)				Ratio DNA nanostructure agarose gel analysis (%)			
	I	II	III	IV	I	II	III	IV
Monomer Sample	100	-	-	-	100	-	-	-
Dimer Sample	23	77	-	-	12	88	-	-
Trimer Sample	17	21	62	-	48	19	33	-
2x2 Sample	44	14	10	33	66	13	3	18

S15: DNA nanostructure yield based on nanopipette repeats

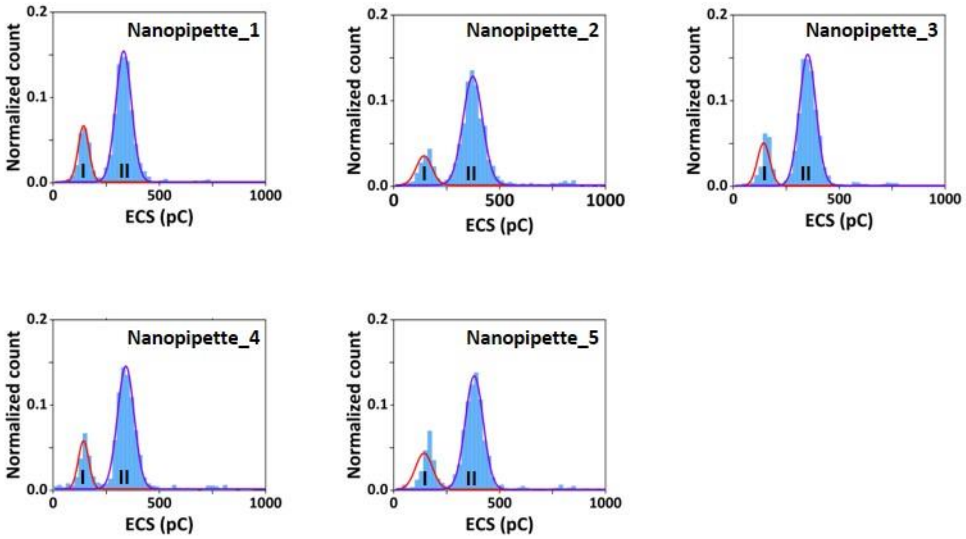

Figure S14: ECS histograms for dimer sample based on recordings obtained from five different nanopipettes measurements.

Table S2: Percentage of dimer DNA nanostructure yield determined from the ECS distributions based on five different nanopipette measurements of the same sample together with the standard deviation.

Nanopipette repeat	Dimer assembly yield quantification			
	**Area ECS peak I**	**Area ECS peak II**	**Dimer nanostructure yield**	**Dimer nanostructure yield ± SD**
**Nanopipette 1**	**4.2**	**14.2**	**77**	
**Nanopipette 2**	**3.2**	**14.7**	**82**	
**Nanopipette 3**	**3.6**	**14.0**	**80**	**79 ± 2**
**Nanopipette 4**	**3.5**	**14.1**	**80**	
**Nanopipette 5**	**4.3**	**14.1**	**77**	

S16: DNA nanostructures design

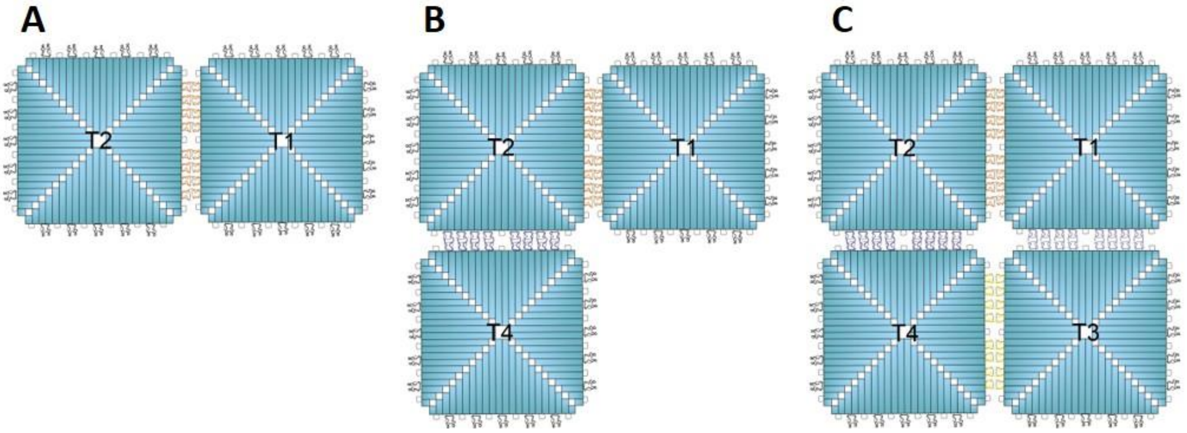

Figure S15: Designs of the higher-order assembly DNA nanostructures indicating the sets of monomer building-blocks used for their assembly: **(A)** - dimer, **(B)** - trimer, **(C)** - 2x2 nanostructure. The sequences for each set of staples are given in the excel file in SI. The notation T1-T4 refers to edge modifications on the monomer nanostructure in order to assemble the higher order DNA nanostructures.

References

1. Plesa, C., and C. Dekker. 2015. Data analysis methods for solid-state nanopores. *Nanotechnology*. 26:084003, doi: 10.1088/0957-4484/26/8/084003.
